# Investigating the Efficacy of Regenerative Therapy With Autogenous Platelet-Rich Plasma in Vertical Bone Defects

**DOI:** 10.7759/cureus.72686

**Published:** 2024-10-30

**Authors:** Tsvetalina Gerova-Vatsova

**Affiliations:** 1 Periodontology and Dental Implantology, Medical University of Varna, Varna, BGR

**Keywords:** biomaterials, periodontal pocket, platelet-rich plasma, regenerative therapy, vertical bone defect

## Abstract

Context and objective

The main goal of periodontal therapy is to remove periodontopathogenic microorganisms and regenerate destructured periodontal tissues. Advances in biomaterials have improved the results of regenerative procedures. However, there is limited data available to determine the best material for vertical bone deformities treated with regenerative treatment. Studies on autogenous platelet-rich plasma (PRP) have been conducted, but it is often combined with other biomaterials. The objective of this study is to examine the exact efficacy of solely administering autogenous PRP in regenerative therapy of vertical bone defects.

Materials and methods

The study was conducted from August 2022 to July 2023 at the Faculty of Dental Medicine, Medical University Varna, BGR, using the University Medical and Dental Center as its base. It includes 12 cases with bipartite, tripartite, or a combination of the listed vertical bone defects. Participating patients met the following criteria: signed informed consent; age between 18 and 65 years; individuals in good health without diagnosed systemic disease, and adequate dental care.

The clinical parameters investigated and recorded immediately before regenerative therapy with autogenous PRP and six months after the surgical intervention were probing depth, margo gingivalis level, clinical attachment level, and cone beam computed tomography (CBCT)-indicators A (the distance from the cemento-enamel junction to the bottom of the bone defect), B (the distance from the cemento-enamel junction to the apex of the bone defect), and C (the width of the defect). The results obtained from the measurements immediately before the regenerative therapy with PRP and six months after the surgical intervention were compared and analyzed.

Results and conclusions

The clinical outcomes for vertical bone defects at the six-month mark following the PRP therapy showed an average 3.83 mm reduction in probing depth, an average 0.08 mm coronal migration of the gingival margin, and an average 3.92 mm gain of the clinical level of attachment. The CBCT showed bone filling, which is supported by the average decrease for points A, B, and C of 1.69 mm, 0.51 mm, and 0.36 mm, respectively. The study's findings primarily illustrate the strong potential of autogenous PRP as an independently applicable substance for regeneration therapy in periodontology.

## Introduction

Following carious lesions, periodontitis is proven to be the next most frequent illness in the oral cavity [1]. It is an infectious condition that causes the structures of the periodontium to sequentially become inflamed and degraded [2]. While there are other risk factors that might impact the initial development, advancement, and forecast of periodontitis, the primary causative element is the particular microorganisms found in dental plaque [3-5].

Alterations in the free gingiva's color, volume, form, and consistency; bleeding upon probing; reduction of clinical attachment level; osseous resorption (supra- and infra-osseous abnormalities); and dental mobility are the most prevalent clinical symptoms of periodontitis [6,7]. The primary objective of periodontal therapy is the removal of periodontopathogenic microorganisms, which are the primary etiological factor, and the regeneration of the destructured periodontal tissues [8,9]. The restoration of the periodontium, the structure that supports the teeth, is known as periodontal regeneration therapy [10].

The results of applying different regenerating procedures have been greatly enhanced throughout time by the development of sophisticated biomaterials. Today, various growth factors, bone healing products, barrier membranes, and combinations of these are employed [11-14]. Platelet-rich material known as autogenous platelet-rich plasma (PRP) is created by carefully processing peripheral blood [15,16]. Plasma and formed components, such as leukocytes, platelets, and erythrocytes, make up peripheral blood. Three different types of granules are seen in platelets: lysosomes, dense granules, and α-granules. Multiple growth factors are released by α-granules with platelet activation and degranulation [17]. Growth factors have been shown to influence chemotaxis, the synthesis of extracellular matrix proteins, and cell migration, proliferation, and metabolic activity. In the context of periodontal regenerative therapy, bioactive materials like PRP are therefore being employed more frequently in addition to well-known biomaterials like bone repair materials and barrier membranes [18-22].

In the last decade, PRP has gained widespread popularity and has found diverse applications in many fields of medicine, namely dental, maxillofacial surgery, dermatology, orthopedics, aesthetic and plastic reconstructive surgery, neurosurgery, etc. [23-25]. Nowadays, PRP is increasingly used as a means of preserving volume from available tissue after tooth extraction [25], as the results obtained with PRP are equivalent to those obtained with other time-proven techniques [26-28].

## Materials and methods

The study was conducted from August 2022 to July 2023 at the Faculty of Dental Medicine, Medical University Varna, BGR, using the University Medical and Dental Center as its base. It includes 12 cases with bipartite, tripartite, or a combination of the listed vertical bone defects. Patients participating in the study met the following criteria: signed informed consent, age between 18 and 65 years, and individuals in good health without diagnosed systemic disease and with adequate dental care.

A systemic and hygiene phase of the therapy sequence was completed beforehand for every patient involved in the trial [29]. The systemic phase's objectives include preserving the patient's and the physician's health by removing or decreasing the impact of systemic factors on periodontal therapy. Consequently, every individual completed a questionnaire regarding his or her medical condition throughout the initial evaluation.

Each patient received a periodontal card during the hygiene phase to be filled out with information about each tooth's prognosis, mobility, furcation involvement, implant presence, probing pocket depth, level of gingival margin, gingival index, and plaque index. An imaging scan called interproximal orthopantomography was performed before beginning periodontal therapy to determine the diagnosis and prognosis of each tooth.

**Figure 1:**
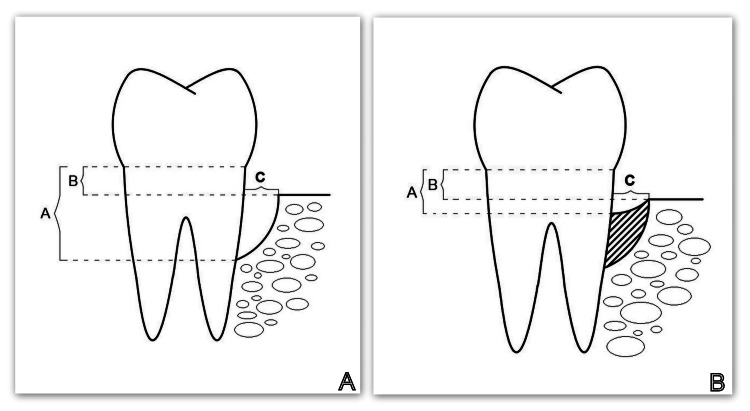
Studied CBCT parameters A: CBCT parameters studied before regenerative therapy; B: CBCT parameters studied six months after regenerative therapy CBCT: Cone beam computed tomography Image credits: Author Gerova-Vatsova T

Following the systemic and hygienic phase of the therapeutic order, each participant had at least one vertical bone defect on the cone beam computed tomography (CBCT) discovered during the re-evaluation stage. Immediately before regenerative therapy with autogenous PRP and six months after the surgical intervention, the investigated clinical parameters of probing pocket depth, level of the gingival margin, clinical attachment level, and CBCT-indicators A (the distance from the cemento-enamel junction to the bottom of the bone defect), B (the distance from the cemento-enamel junction to the apex of the bone defect), and C (the width of the defect) were recorded (Figure 1).

**Figure 2:**
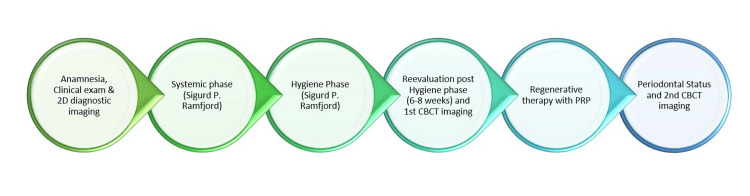
Flowchart explaining the sequence of the study for each case Systemic phase and hygienic phase: "A Rational Plan for Periodontal Therapy," Ramfjord SP [29] Image credits: Author Gerova-Vatsova T

Six months after the regenerative therapy with PRP, a new periodontal status was recorded, and the clinical parameters were evaluated. A new CBCT examination was ordered, and parameters A, B, and C were reassessed (Figure 2).

The study compared and analyzed metrics from pre-regenerative therapy with PRP and six months post-surgical intervention. The statistical methods used to analyze and organize the results obtained were analysis of variance, one-sample t-test, paired-samples t-test, and one-way ANOVA.

Protocol for obtaining PRP

In addition to the laboratory centrifuge ЕВA20-HettichLab (Hettich, Tuttlingen, DEU), the subsequent laboratory supplies are necessary for the acquisition of PRP: an 8-mL vacutainer with a biocompatible inert gel (erythrocyte separation and anticoagulant action) and some single-use materials. Calcium gluconate and calcium gluconicum ampule 10% are utilized for the product's final activation.

Venipuncture is conducted using a needle with a diameter of no less than 22G which inhibits platelets from activating earlier. Each patient had 8 mL of blood extracted. The latter is processed within one hour after its extraction during the surgical procedure. The extracted blood is transported into the vacutainer. The last one is subsequently agitated to amalgamate the blood with the anticoagulant.

The vacutainer is positioned in the laboratory centrifuge. A fundamental element for its operation is weight adjustment, achieved by parallel positioning the test tubes and ensuring the same volumes within them. Centrifugation is conducted at ambient thermal conditions of 20°C to 22°C in two phases. During the initial separation centrifugation, a centrifugal force of 1150xg is applied for 10 minutes, followed by a second concentration centrifugation with a force of 350xg for five minutes. We utilized the technical parameters of the centrifuge to derive the cycles employed in the initial and secondary centrifugation.

**Figure 3:**
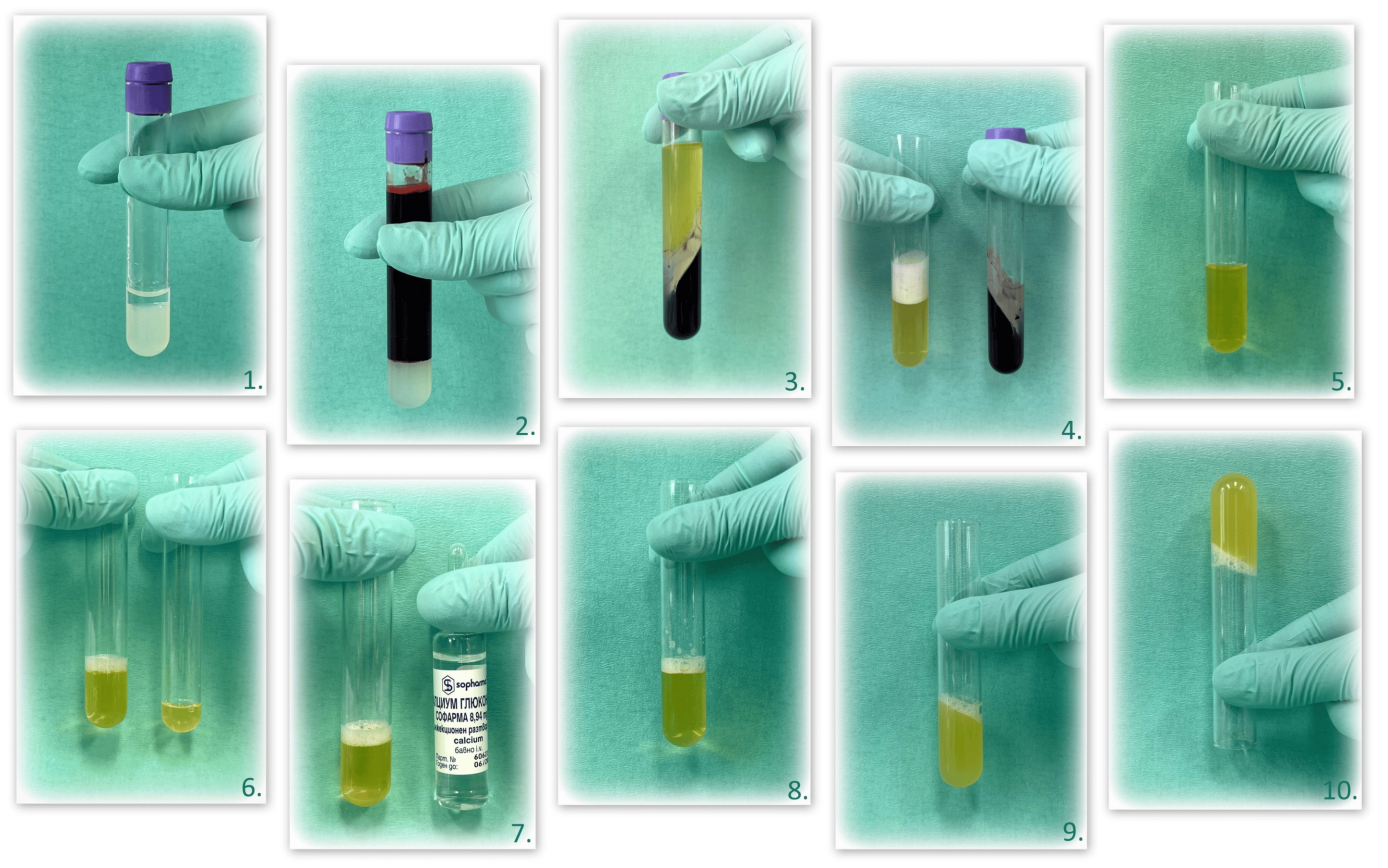
Protocol for obtaining PRP 1: Vacutainer with a biocompatible inert gel (erythrocyte separation and anticoagulant action); 2: Around 8 mL of venous blood in the same vacutainer; 3: Formation of three layers after the first separation centrifugation; 4: Transferred buffy coat (left) and PPP from the vacutainer (right); 5: Tube after second concentration centrifugation; 6: The most superficial part of the PPP is removed (right) from the rest together with the buffy coat (left) layer (with a total volume of about 3 mL); 7: 3 mL biological material (buffy coat and PPP on the left) and calcium gluconate activator (right); 8: 3 mL including buffy coat and PPP homogenized with 1 ml of calcium gluconate; 9 and 10: PRP after 20 minutes PRP: Platelet-rich plasma, PPP: Platelet-poor plasma

Platelet-rich plasma is derived via the 'buffy coat' technique, wherein the initial centrifugation occurs at elevated relative centrifugal force (RCF) levels. Consequently, three distinctly observable layers are formed: the first, at the bottom, is abundant in erythrocytes; the middle one, containing white blood cells, is referred to as the buffy coat; and the third, the uppermost layer, is the platelet-poor plasma (PPP). Following the initial centrifugation, the top two layers (buffy coat and PPP) are extracted and then moved to a sterile tube for the following centrifugation. Following the second centrifugation, below the buffy coat layer, a minimal number of erythrocytes may be observed, and a layer of plasma once more on top. The layer of the PPP is excised, and the remaining material, along with the buffy coat layer (approximately 3 mm in total volume), is aspirated. This material is then transferred to a sterile tube, where it is subsequently activated with calcium gluconate. Of the acquired plasma, 3 mL is positioned in a tube, followed by the addition of 1 mL of calcium gluconate. The mix is homogenized and allowed to settle at ambient temperature for 20 minutes (Figure 3). The resultant substance, which looks and behaves like gel, is utilized for regenerative therapy of superficial vertical defects or to perform guided tissue regeneration of substantial vertical defects.

Clinical protocol for regenerative therapy with autogenous PRP in vertical bone defects

**Figure 4:**
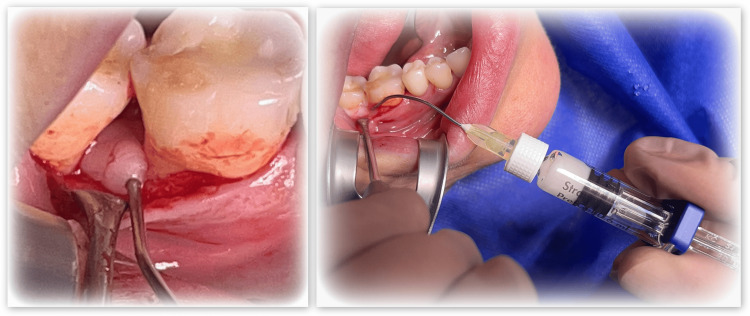
Conditioning the root surface with Straumann® PrefGel

The surgical area is treated locally with anesthesia utilizing one Septanest (Septodont, Saint-Maur-des-Fossés, FRA). An intrasulcular incision with a width of up to two teeth is executed using a #15 blade scalpel. This is followed by the reflection of a mucoperiosteal flap utilizing an elevator. The infected tissue in the defect is excised using scaling and root planing with universal curettes. The surgical area is irrigated with saline solution. The surface of the root is treated for two minutes with Straumann® PrefGel (Straumann Group, Basel, CHE) (Figure 4).

**Figure 5:**
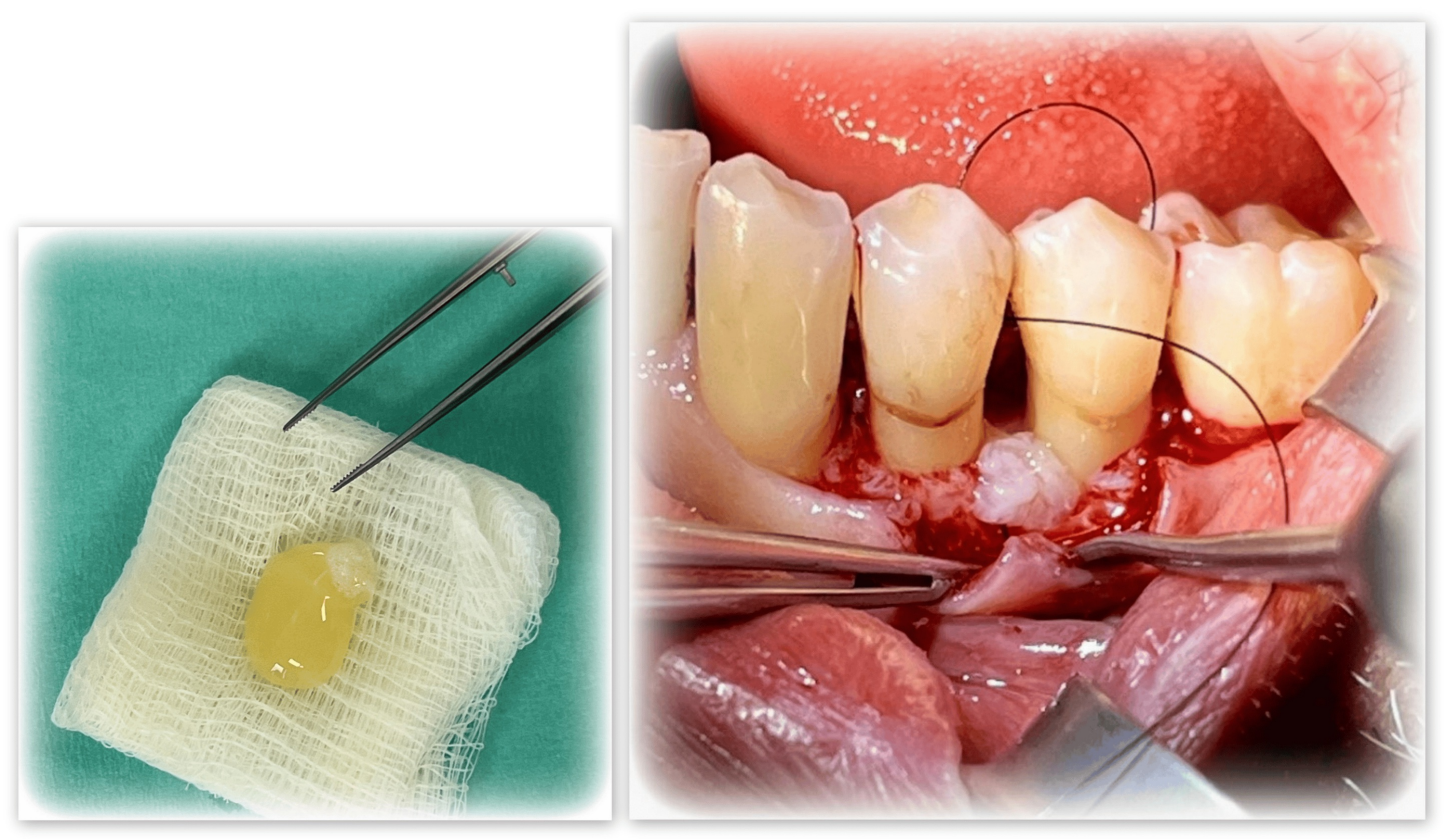
Administering the PRP into the vertical bone defect PRP: Platelet-rich plasma

Following extensive cleansing with ethylenediaminetetraacetic acid (EDTA) gel and sodium chloride, the earlier acquired PRP was administered into the vertical bone defect (Figure 5). This was succeeded by the suturing of the flap (Dafilon; B. Braun, Melsungen, DEU).

Post-surgical therapy was comprised of an antibiotic, a nonsteroidal anti-inflammatory drug, and a mouthwash (with chlorhexidine). A meeting was scheduled for a follow-up evaluation and to take out the sutures two weeks after the surgery.

## Results

Parameters, both clinical and paraclinical, that were measured immediately before the surgical intervention of the patients (zero months) were compared with those measured in the sixth month after the regenerative therapy with PRP. The results are summarized below.

Probing pocket depth

**Figure 6:**
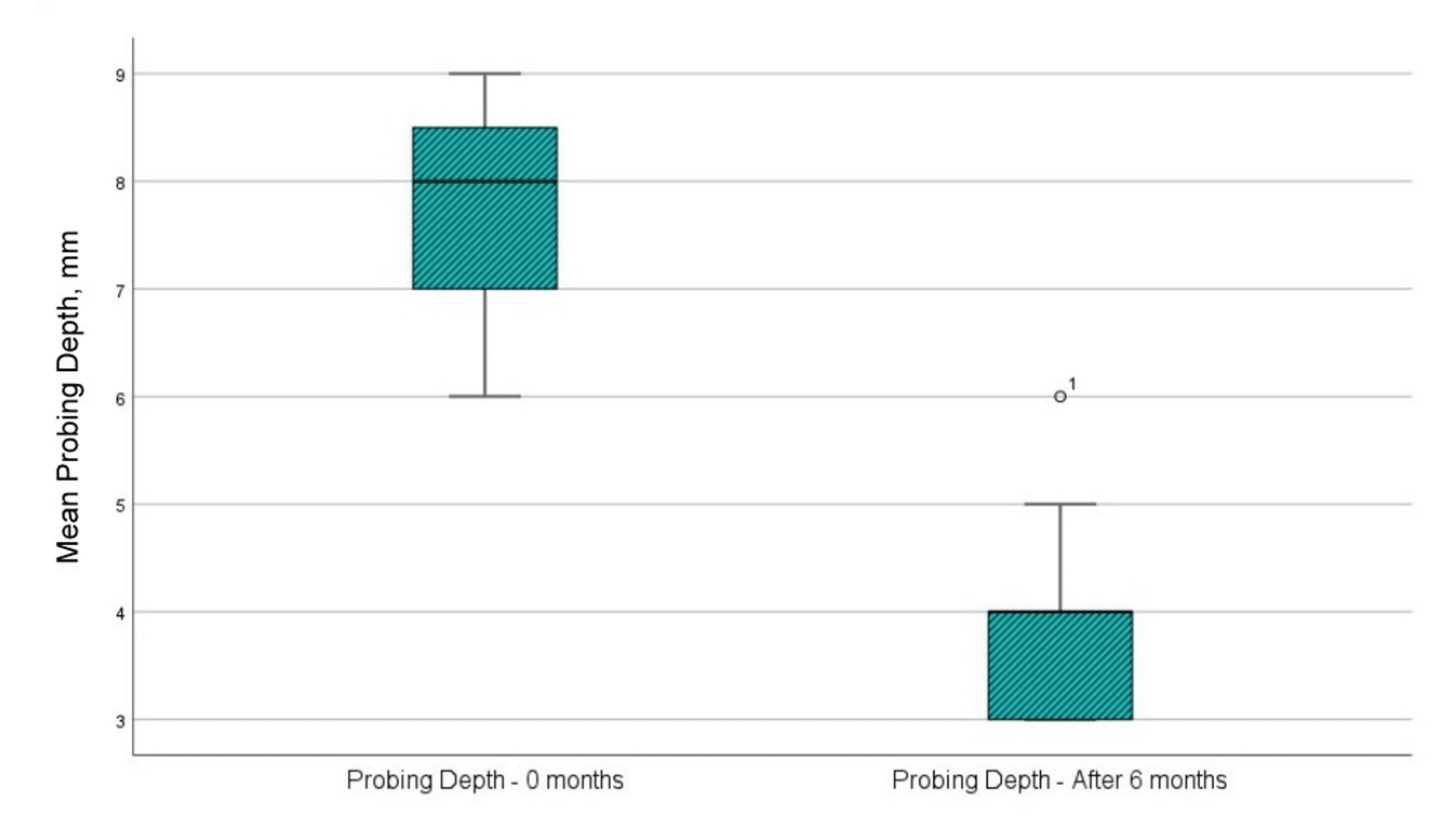
Mean values of the probing depth at the time of examination before the surgical intervention and six months after regenerative therapy

From Figure 6 and Table 1, we can conclude that the mean value of the probing depth at the examination immediately before the surgical intervention was 7.75 mm, while the same mean value in the sixth month after the regenerative therapy with PRP had decreased to 3.92 mm. Analysis of the depth of probing indicators at zero months and six months after regenerative therapy with PRP showed that the mean value of this indicator was reduced by 3.833 mm. Analysis by t-test shows that the difference in the depth of probing indicator is statistically significant, as p<0.001, which is <0.05; the upper and lower limits of significance do not cross 0, and the t-value is greater than 1.796, which is the reference value at 11 dF. The results are presented in Table 2.

**Table 1:** Paired samples statistics with mean values and deviation CBCT: Cone beam computed tomography

Parameter	Time of evaluation	Mean	N	Standard deviation	Standard error mean
Probing depth	0 months	7.75	12	0.965	0.279
	6 months	3.92	12	0.900	0.260
Level of margo gingivalis	0 months	0.08	12	0.996	0.288
	6 months	0.17	12	0.835	0.241
Clinical attachment level	0 months	-7.67	12	1.497	0.432
	6 months	-3.75	12	1.357	0.392
CBCT parameter А	0 months	6.37	12	0.793	0.229
	6 months	4.68	12	1.105	0.319
CBCT parameter В	0 months	3.19	12	0.864	0.250
	6 months	2.69	12	0.593	0.171
CBCT parameter С	0 months	2.37	12	0.450	0.130
	6 months	2.01	12	0.770	0.222

**Table 2:** Mean comparison and significance test CBCT: Cone beam computed tomography, df: Degrees of freedom

Parameter	Paired differences			95% Confidence interval of the difference				Significance	
	Mean	Standard deviation	Standard error mean	Lower	Upper	t-value	df	One-sided p-value	Two-sided p-value
Probing depth	3.833	0.718	0.207	3.377	4.289	18.501	11	<0.001	<0.001
Level of margo gingivalis	-0.083	0.996	0.288	-0.716	0.550	-0.290	11	0.389	0.777
Clinical attachment level	-3.917	0.996	0.288	-4.550	-3.284	-13.619	11	<0.001	<0.001
CBCT parameter A	1.689	0.759	0.219	1.207	2.171	7.711	11	<0.001	<0.001
CBCT parameter B	0.506	0.481	0.139	0.201	0.811	3.646	11	0.002	0.004
CBCT parameter C	0.356	0.697	0.201	-0.087	0.799	1.767	11	0.052	0.105

Level of gingival margin

**Figure 7:**
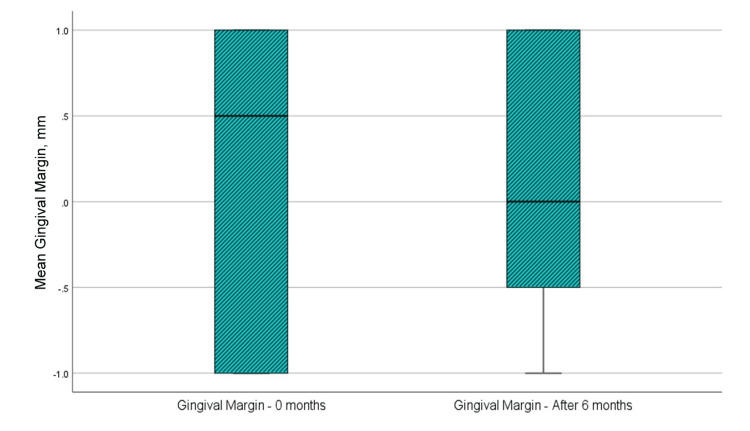
Mean values of the level of margo gingivalis at the time of examination before surgical intervention and six months after regenerative therapy

From Figure 7 and Table 1, it is found that the mean value of the level of margo gingivalis at the time of examination immediately before surgical intervention was 0.08 mm, while the same mean value six months after regenerative therapy with PRP was 0.17 mm. Analyzing the margo gingivalis level parameter at zero months and six months after regenerative therapy with PRP, it is visible that the gingival margin has migrated coronally relative to the cemento-enamel junction by 0.083 mm on average. The analysis using the t-test showed that the difference in the margo gingivalis level parameter was not statistically significant because, although p=0.389, slightly less than 0.05, the upper and lower limits of significance crossed 0 and the t-value was less than 1.796, which is the reference value at 11 df. The results are presented in Table 2.

Clinical attachment level

**Figure 8:**
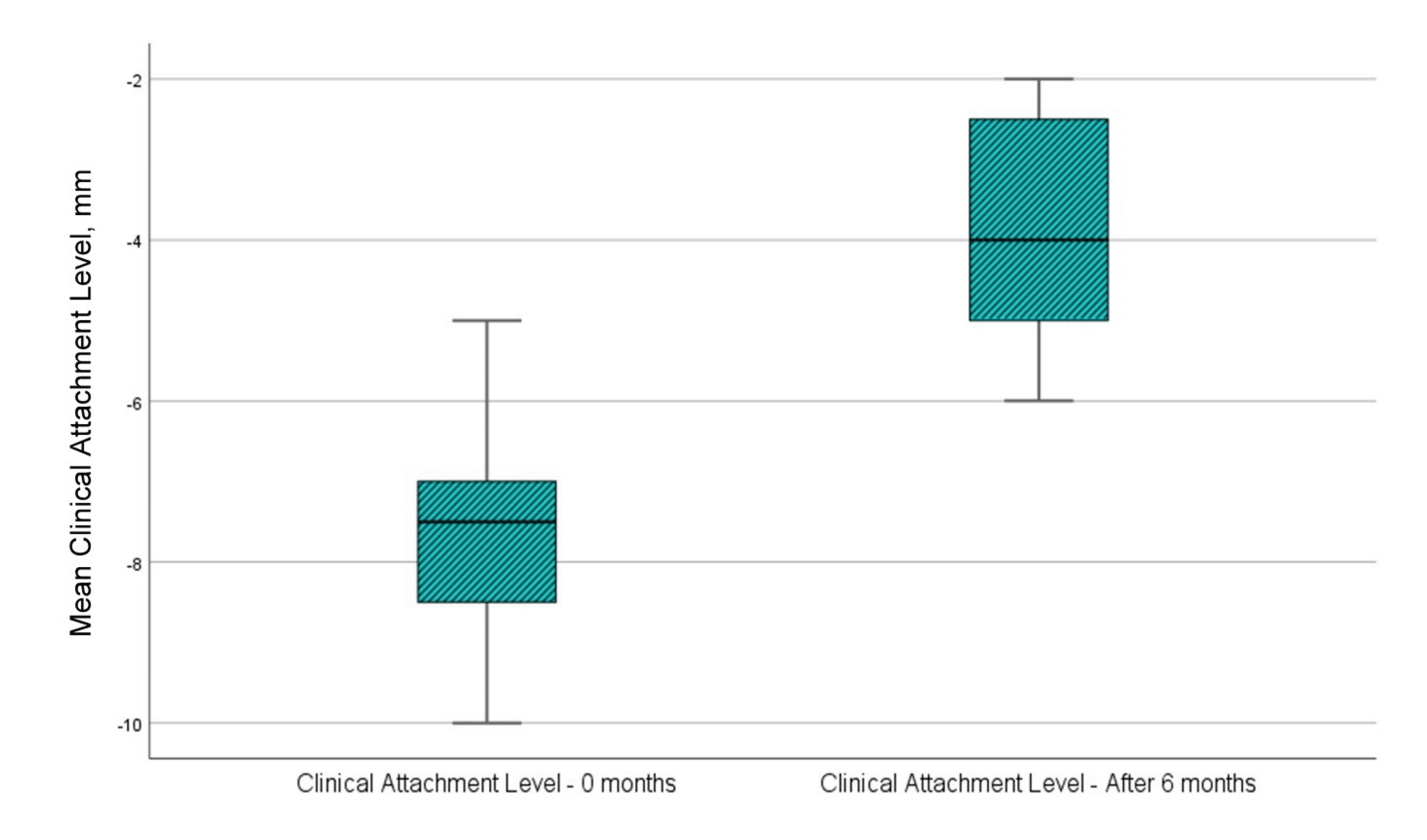
Mean values of the clinical attachment level at the time of examination before surgical intervention and six months after regenerative therapy

Figure 8 and Table 1 show that the mean clinical attachment level at the examination immediately before the surgical intervention was 7.67 mm, whereas the same mean value in the sixth month after regenerative therapy with PRP was 3.75 mm. Examining the clinical level of attachment indicators at zero months and six months after regenerative therapy with PRP, it is visible that the distance from the gingival margin to the bottom of the pocket has decreased by an average of 3.917 mm, i.e., a gain in clinical level of attachment has happened. The t-test analysis indicated that the difference in the parameter of the clinical level of attachment was statistically significant because p<0.001, which is <0.05, the upper and lower significance limits did not cross 0, and the t-value was greater than 1.796, which is the reference value at 11 df (Table 2).

CBCT parameter A (the distance from the enamel cement barrier to the bottom of the bone defect)

**Figure 9:**
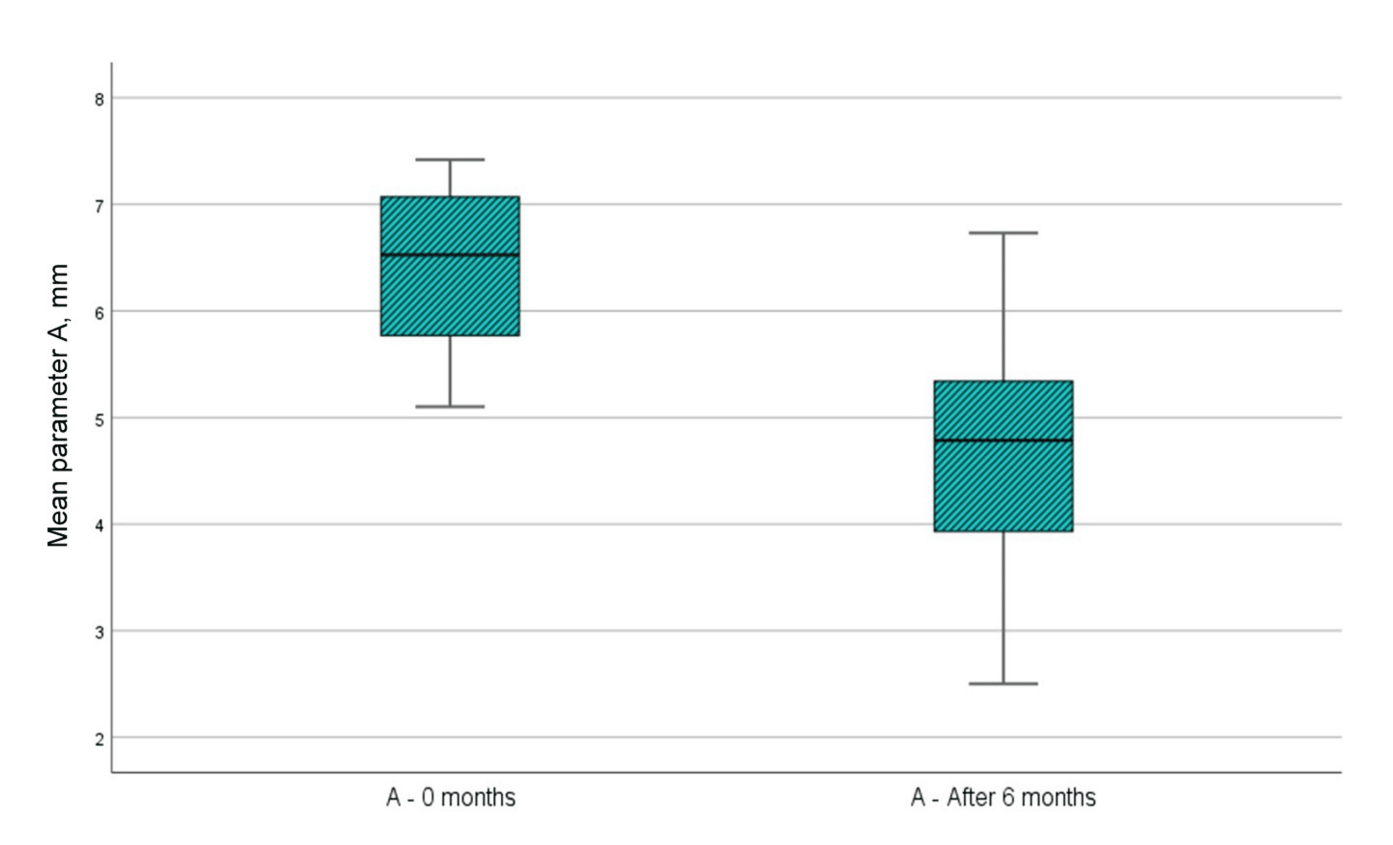
Mean values of parameter A at the time of examination before surgical intervention and six months after regenerative therapy

Figure 9 and Table 1 show that the mean value of the paraclinical index A at the time of examination immediately before surgical intervention was 6.37 mm, while the same mean value in the sixth month after regenerative therapy with PRP was 4.68 mm. Analyzing the indicators of parameter A at zero months and six months after regenerative therapy with PRP, it is visible that the distance from the cemento-enamel junction to the bottom of the bone defect assessed on CBCT has diminished by a median of 1.689 mm, i.e., bone filling of the defect has occurred. The analysis by t-test showed that the difference in parameter A was statistically significant as p<0.001, which is <0.05, the upper and lower significance limits did not cross 0, and the t-value was greater than 1.796, which is the reference value at 11 dF (Table 2).

CBCT parameter B (distance from the enamel cement barrier to the apex of the bone defect)

**Figure 10:**
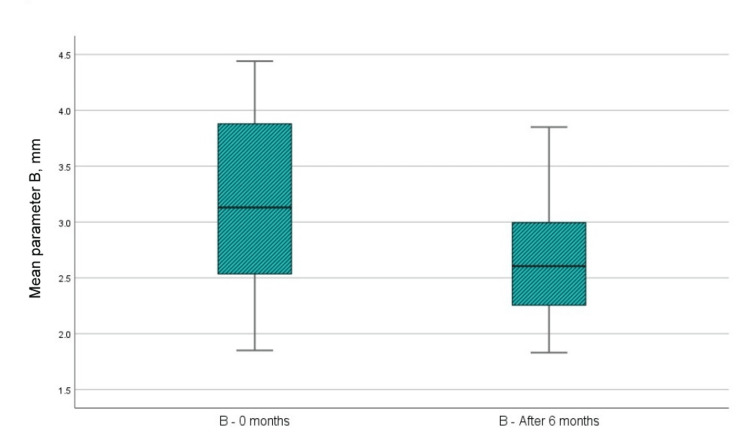
Mean values of parameter B at the time of examination before surgical intervention and six months after regenerative therapy

Figure 10 and Table 1 show that the mean value of the paraclinical index B at the time of examination immediately before surgical intervention was 3.19 mm, while the same mean value in the sixth month after the regenerative therapy with PRP was 2.69 mm. In the analysis of parameter B indicators at zero months and six months after regenerative therapy with PRP, it is visible that the distance from the cemento-enamel junction to the apex of the bone defect assessed on the CBCT has diminished by a median of 0.506 mm, i.e., bone filling of the defect has occurred. Analysis by t-test showed that the difference in parameter B was statistically significant, as p<0.001, which is <0.05, the upper and lower limits of significance did not cross 0, and the t-value was greater than 1.796, which is the reference value at 11 df (Table 2).

CBCT parameter C (width of the bone defect)

**Figure 11:**
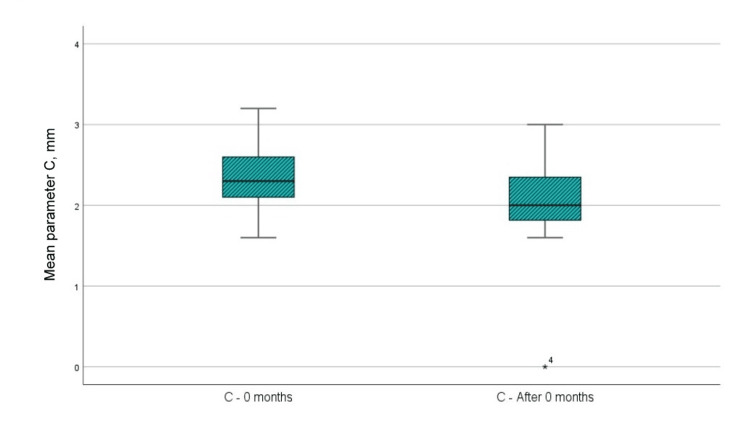
Mean values of parameter C at the time of examination before surgical intervention and six months after regenerative therapy

Figure 11 and Table 1 show that the mean value of paraclinical index C at the time of examination immediately before surgical intervention was 2.037 mm, while the same mean value in the sixth month after the regenerative therapy with PRP was 2.01 mm. When analyzing parameter C indicators at zero months and at six months after regenerative therapy with PRP, it is visible that the width of the bone defect has diminished by an average of 0.356 mm, i.e., bone filling of the defect has occurred. The analysis using a t-test shows that the difference in parameter C is not statistically significant as p=0.052 which is >0.05, the upper and lower limits of significance crossed 0, and the t-value is less than 1.796, which is the reference value at 11 df (Table 2).

## Discussion

**Figure 12:**
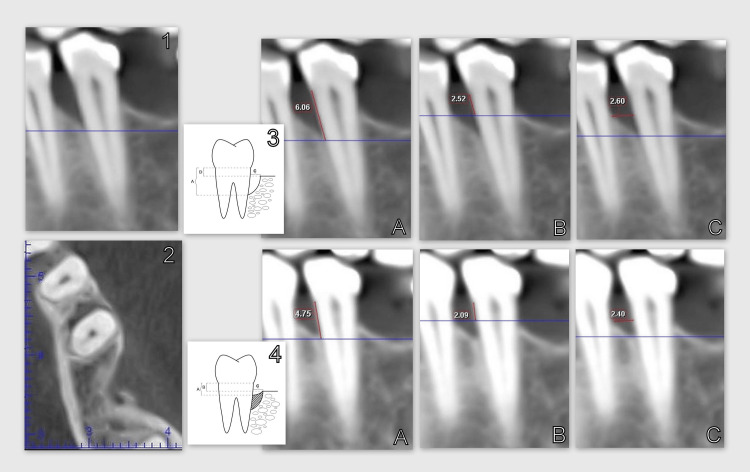
The CBCT images before and after regenerative therapy with PRP 1: Coronal plane of the vertical defect on CBCT; 2: Transversal plane of the vertical defect on CBCT; 3: CBCT parameters studied before regenerative therapy; 3A: Parameter A (zero months); 3B: Parameter B (zero months); 3C: Parameter C (zero months); 4: CBCT parameters studied six months after regenerative therapy; 4A: Parameter A (six months); 4B: Parameter B (six months); 4C: Parameter C (six months) CBCT: Cone beam computed tomography

The clinical outcomes observed six months post-regenerative therapy utilizing PRP in vertical bone defects indicated an average reduction in probing depth of 3.83 mm, a coronal shift of the gingival margin averaging 0.08 mm, and an average clinical attachment level gain of 3.92 mm. In this study, CBCT was used to evaluate the regeneration process. The method allows for volumetric assessment of the alveolar bone with high resolution and low radiation exposure [30]. The bone filling is evident in the CBCT and is corroborated by the following measurements: an average reduction of 1.69 mm in parameter A; an average reduction of 0.51 mm in parameter B; and an average reduction of 0.36 mm in parameter C. Figure 12 showcases the metrics of a random case out of the 12 cases.

Analysis of the clinical attachment level indicators at zero months and six months post-regenerative therapy with PRP reveals a significant reduction in the distance from the gingival margin to the base of the pocket, averaging 3.917mm, indicating the attainment of a clinical level of attachment. Simultaneously, an analysis of parameter A indicators at zero months and six months post-regenerative therapy with PRP reveals a reduction in the distance from the cemento-enamel junction to the bottom of the bone defect, as assessed by CBCT, averaging 1.689 mm. A higher proportion of soft tissue acquired compared to regenerated bone was noted (3.917 mm minus 1.689 mm equals 2.228 mm).

The evaluation of the study results proves that the methodology of regenerative therapy with PRP would be competitive with all methodologies accepted as the gold standard in periodontics. Currently, there is a lack of sufficient publications addressing the efficacy of PRP as a sole-administered material in periodontal therapy, aside from a 2018 study by Jalaluddin et al. [19], which compared the regenerative efficiency of PRP (alone) with bone grafts in vertical bone defects. The researchers determined that the two groups exhibited encouraging outcomes following regenerative therapy.

The challenge that was encountered during this study was mainly the timing of PRP production and operational area preparation time. The limitation of the present study, which may have influenced the results slightly, was the degree of compliance with all instructions to maintain excellent oral hygiene by the subjects during these six months. At this stage, the results of the clinical study need to be followed up over time to assess and investigate the results in a larger number of cases and over a longer period. Only then will regenerative therapy with PRP alone be accepted as an established method.

## Conclusions

The study findings primarily illustrate the strong potential of PRP as an independently applicable material for regeneration therapy of vertical bone defects. The methodology of regenerative therapy with PRP has a facilitated surgical protocol compared to the methodology of guided tissue regeneration, which would make PRP-regenerative therapy a preferred method to work with in clinical practice. Additionally, post-surgical issues occur less frequently in comparison to those of guided tissue regeneration. The extremely low financial cost of this method should also be highlighted here as an advantage. The main disadvantage of regenerative therapy with PRP lies in the fact that the process of producing the PRP is more work-intensive and time-consuming and is linked to potential human mistakes.
